# Kindlin-2 deficiency induces fatal intestinal obstruction in mice

**DOI:** 10.7150/thno.46553

**Published:** 2020-05-15

**Authors:** Xiaokun He, Jiagui Song, Zeyu Cai, Xiaochun Chi, Zhenbin Wang, Decao Yang, Sian Xie, Jing Zhou, Yi Fu, Wei Li, Wei Kong, Jun Zhan, Hongquan Zhang

**Affiliations:** 1 Department of Human Anatomy, Histology and Embryology, Key Laboratory of Carcinogenesis and Translational Research, Ministry of Education, and State Key Laboratory of Natural and Biomimetic Drugs, Peking University Health Science Center, Beijing 100191, China; 2 Department of Physiology and Pathophysiology, School of Basic Medical Sciences, Peking University Health Science Center, Beijing 100191, China; 3 Department of Surgery and Interdepartmental Program in Vascular Biology and Therapeutics, Yale University School of Medicine, New Haven, Connecticut, USA

**Keywords:** Kindlin-2, Smooth muscle structure, Smooth muscle contraction, Intestinal obstruction

## Abstract

**Rationale**: Smooth muscle-motility disorders are mainly characterized by impaired contractility and functional intestinal obstruction. Some of these cases are caused by genetic mutations of smooth muscle genes ACTA2, ACTG2, MYH11, MYLK and LMOD1. Still the etiology is complex and multifactorial and the underlying pathology is poorly understood. Integrin interaction protein Kindlin-2 is widely expressed in striated and smooth muscle cells (SMC). However, the function of Kindlin-2 in the smooth muscle remains elusive.

**Methods**: We generated two mouse models using different cre promoter transgenic mice, Kindlin-2^fl/fl^ SM22α-cre+ (cKO mice) and Kindlin-2^fl/fl^; MYH-cre+ (iKO mice). Embryos and adult tissues were prepared for hematoxylin and eosin (H&E) staining, immunohistochemistry (IHC) and terminal deoxynucleotidyl transferase dUTP nick-end labeling (TUNEL) apoptosis assay. We investigated ultrastructure changes of mouse smooth muscle using transmission electron microscopy (TEM) and measured smooth muscle contractile force in mounting aortic and intestinal rings using the multiwire myograph system (DMT 620M). In addition, cell traction force microscopy (CTFM) was applied to observe the functional change of primary SMC after Kindlin-2 depletion by RNAi.

**Results**: Depletion of Kindlin-2 encoding gene Fermt2 in embryonic smooth muscles leads to apoptosis, downregulates the key components of SMC, impairs smooth muscle development, and finally causes embryonic death at E14.5. Tamoxifen-induced Kindlin-2-specific knockout in adult mouse smooth muscle showed decreased blood pressure, intestinal hypoperistalsis, and eventually died of intestinal obstruction. Kindlin-2 depletion also leads to downregulated Myh11, α-SMA, and CNN, shortened myofilament, broken myofibrils, and impaired contractility of the smooth muscles in iKO mice. Mechanistically, loss of Kindlin-2 decreases Ca2^+^ influx in primary vascular smooth muscle cells (PVSMC) by downregulating the expression of calcium-binding protein S100A14 and STIM1.

**Conclusion**: We demonstrated that Kindlin-2 is essential for maintaining the normal structure and function of smooth muscles. Loss of Kindlin-2 impairs smooth muscle formation during embryonic development by inducing apoptosis and jeopardizes the contraction of adult smooth muscle by blocking Ca^2+^ influx that leads to intestinal obstruction. Mice with Kindlin-2 depletion in adult smooth muscle could be a potent animal model of intestinal obstruction for disease research, drug treatment and prognosis.

## Introduction

Smooth muscle cells (SMCs) express cytoskeletal and contractile proteins, such as α-SMA, smooth muscle myosin heavy chain (SMMHC, also known as myosin heavy chain 11, MYH11), calponin (CNN), and smooth muscle protein 22-alpha (SM22α) 1. Contraction of SMCs results from an elaborate molecular process. G protein‐mediated activation of phospholipase C β and generation of the secondary messenger 1,4,5-trisphosphate causes Ca^2+^ release from the sarcoplasmic reticulum (SR) in SMCs. Ca^2+^ can also enter SMCs through voltage-dependent calcium channels and store-operated calcium entry (SOCE), both of which are activated by Ca^2+^ depletion from intracellular stores. Stim1 was previously identified as an SR Ca^2+^ sensor, and Ca^2+^ depletion from the SR caused Stim1 to accumulate at SR-plasma membrane (PM) junctions where it can trap and activate Orai channels diffusing towards the closely apposed PM 2. An increase in cytosolic free Ca^2+^ triggers calmodulin‐dependent activation of myosin light chain kinase (MLCK), which subsequently phosphorylates the 20-kDa regulatory myosin light chain (MLC20) to form actin-myosin cross-bridges, leading to muscle contraction 3-5. Loss of any of the proteins involved in this process is likely to affect cell physiology, resulting in impaired contractility and smooth muscle-motility disorders such as chronic intestinal pseudo-obstruction (CIPO) and megacystis microcolon intestinal-hypoperistalsis syndrome (MMIHS). CIPO may be congenital or acquired secondary to other disorders. One-third of congenital CIPO cases are smooth muscle myopathic origin, and proper diagnosis and treatment of CIPO are difficult because the underlying pathology is poorly understood 6, 7. MMIHS is a rare congenital disease of the visceral organs, mainly characterized by bladder distension and functional intestinal obstruction 8. Lots of smooth muscle-related genetic mutations were identified to link with MMIHS, such as heterozygous mutations in MYH11 and ACTA2 9-11, and homozygous mutations in smooth muscle gamma-2 actin (*ACTG2*), MYH11, myosin light chain kinase (MYLK), and leimodin 1(LMOD1) 12-16. Although the identification of pathogenic variants in these genes explains approximately 90% of all MMIHS, for some affected individuals, the causative mutation and associated gene have yet to be identified.

In situ hybridization experiments showed that Kindlin-2 is preferentially expressed in striated and smooth muscle cells (SMCs) 17. The Kindlin family represents a class of focal adhesion proteins that are implicated in integrin activation, which is essential for dynamically linking the extracellular environment and cytoskeletal/signaling networks. They comprise three evolutionarily conserved members, namely Kindlin-1, Kindlin-2, and Kindlin-3, which share considerable sequence and structural similarities. Kindlins have a bipartite four point one protein, ezrin, radixin, moesin (FERM) domain that is interrupted by a pleckstrin homology domain, can bind directly to various classes of integrins, and participates in inside-out integrin activation. Loss-of-function mutations in Kindlin-1 and Kindlin-3 cause Kindler Syndrome and leukocyte adhesion deficiency-III syndrome, respectively 18.

Kindlin-2 (also known as MIG-2 and Fermt2), is physically attached to the integrin tail, via β1 subunit, and plays a central role in integrin regulation, activation, clustering and signaling 19, 20. Apart from the pivotal role of Kindlin-2 in tumor progression 21, 22, Kindlin-2 plays critical role in muscle development and in myogenesis 23, 24. Further, Kindlin-2 regulates myogenin (MyoG) expression in C2C12 cells (immortalized mouse myoblast cell line) through Wnt/β-catenin signaling, independent of its integrin binding, therefore playing a role in muscle differentiation. In addition, Kindlin-2, as an essential component of the intercalated disc and Z disc 25, is necessary for the cytoskeletal organization at sites of membrane attachment and is required for vertebrate myocardial cell formation and function 26.

Loss of Kindlin-2 in mice results in embryonic lethality at E7.5 before cardiogenesis 26, which is caused by severe detachment of the endoderm and epiblast from the basement membrane 27. The induced deletion of Kindlin-2 has already been found to lead to cardiac dysfunction in mice by our lab 28. Morpholino-based knockdown of a Kindlin-2 homolog in zebrafish (z-Kindlin-2) resulted in severe abnormalities in heart development. Morphant hearts were hypoplastic and dysmorphic and exhibited significantly reduced ventricular contractility 26. Kindlin-2 has also been found to play critical angiogenic roles in mice and zebrafish. Heterozygous knockout mice were viable and, when challenged, showed significantly reduced angiogenesis in growing tumors and Matrigel implants 29. Specifically, differentiating myocytes lacking Kindlin-2 were unable to elongate and failed to fuse into multinucleated myotubes 30. As Kindlin-2 is fundamental for integrin complex stability and for myogenesis, it is potentially essential for maintaining the normal structure and function of smooth muscle and we hypothesis that depletion of Kindlin-2 could induce CIPO & MMIHS in mice.

In this study, we utilized knockout mice to model the in vivo effects of smooth muscle Kindlin-2 depletion. SM22α is a putative calcium-binding protein that is expressed in cardiac, smooth, and skeletal muscle cell lineages during mouse embryogenesis and in adult SMCs 31. The transgenic mice used in this study expressed Cre recombinase under the control of the mouse SM22α promoter. Cre recombinase activity was observed in vascular SMCs of the aorta, gastrointestinal tract, and uterus. When crossed with a strain containing a *loxP* site-flanked sequence of interest, Cre-mediated recombination caused depletion of the flanked sequence in vascular SMCs. This strain represents an effective tool for generating tissue-specific targeted mutants and should be useful for studying smooth muscle diseases.

## Methods

### Mice and Genotyping

All animal experiments were approved by the Peking University Animal Care and Use Committee.

The Kindlin-2 floxed mice were developed by our lab 32, exons 5 and 6 are flanked by loxP sites. SM22α-cre transgenic mice were bought from Nanjing Biomedical Research Institute of Nanjing University. Kindlin-2 floxed (Kindlin-2^fl/fl^) mice were crossed with SM22α-cre mice to generate Kindlin-2 heterozygous mice (Kindlin-2^fl/wt^;SM22α-cre+), which were further backcrossed with Kindlin-2^fl/fl^ mice to generate smooth muscle-specific Kindlin-2 depletion mice. MYH-cre mice, or synonym of MyH11-ERT-Cre mice, were generously provided by Wei Li lab (24). Only male mice inherit the MYH-cre allele, and thus any experiments performed using this mouse model were done so using male mice. MYH-cre mice were bred with the Kindlin-2 flox mice to obtain the Kindlin-2^fl/fl^;MYH-cre+ mice. All male mice (Kindlin-2^fl/fl^;MYH-cre-, Kindlin-2^fl/wt^;MYH-cre+ and Kindlin-2^fl/fl^;MYH-cre+) were treated with tamoxifen (Sigma-Aldrich, T5648) via intraperitoneal injection (1 mg/day; 5 consecutive days; 10 mg/ml of tamoxifen in corn oil) starting at 6-8 weeks of age.

Genotyping was performed via PCR using primers: Kindlin-2: forward 5'- TAC AGG TGG CTG ACA AGA TCC -3', reverse 5'- GTG AGG CTC ACC TTT CAG AGG -3'; SM22α-cre: forward 5'- GCG GTC TGG CAG TAA AAA CTA TC -3', reverse 5'- GTG AAA CAG CAT TGC TGT CAC TT -3'; MYH-cre: SMWT1 5'-TGA CCC CAT CTC TTC ACT CC-3' SMWT2 5'-AAC TCC ACG ACC ACC TCA TC-3' phCREAS1 5'-AGT CCC TCA CAT CCT CAG GTT-3'.

### Western Blot Assay

Fresh tissues were rinsed in ice-cold 0.9% sodium chloride and homogenized in RIPA buffer (50 mM Tris-HCl, 150 mM NaCl, pH 7.4, 0.5% sodium deoxycholate, 1% NP-40, and 0.1% SDS) (Applygen, Beijing, China) with protease inhibitors cocktail by tissue homogenizer (ULTRA-TURRAX® T10 Basic Disperser, IKA® Works, Germany), and centrifuged at 12,000 rpm for 30 min at 4°C to acquire clear lysates. Then protein concentrations were detected by bicinchoninic acid protein assay kit (Applygen, Beijing, China). Protein lysates (40 mg) was loaded in 10% sodium dodecyl sulphate-polyacrylamide gel electrophoresis (SDS-PAGE) and transferred to polyvinylidene fluoride (PVDF) membrane. The membranes were blocked in 5% skim milk at room temperature for 1 h and incubated with specific primary antibodies overnight at 4°C. Antibodies used in this study were: Kindlin-2 (Sigma, K3269, 1:500 dilution); Myh11 (Abcam, ab125884, 1:500 dilution); α-SMA (Abcam, ab5694, 1:500 dilution); CNN (Abcam, ab46794, 1:1000 dilution); Stim1 (ABclonal, A7411, 1:1000 dilution); Actin (Santa Cruz, sc-58673, 1:500 dilution). After washed in TBST for three times, the membranes were incubated with secondary antibodies for 1 h at room temperature. The immobilized antibodies were detected with the Super Signal Chemiluminescence kit (Thermo Fisher Scientific) by enhanced chemoluminoscence (Pierce Chemical Co, Rockford, IL, USA).

### Histology and Immunohistochemistry

Embryos and adult tissues were fixed with 4% buffered formalin (Fisher), paraffin embedded, and sectioned at 5μm before staining. The prepared sections were stained with H&E to examine the structure or with immunohistochemistry for the detection of different proteins. Briefly, the sections were blocked by immersion in 3% hydrogen peroxide (H_2_O_2_) solution for 30 min. After washing in phosphate buffer saline (PBS, pH 7.6) for 10 min, antigen-retrieval was performed by heating slides in 0.01 M citrate buffer (pH 6.0) to approximate 100°C for 15-20 min by water-bath heating in a water bath kettle. Allowing slides and citrate buffer cooled at room temperature. Then 10% normal goat serum was used to block the non-specific binding of antibodies for 30 min. The sections were probed with anti- Kindlin-2(Sigma, K3269, 1:500 dilution), α-SMA (Abcam, ab5694, 1:500 dilution) or caspase-3 (Abcam, ab4051, 1:400 dilution) overnight, washed in PBS three times and then incubated with biotinylated IgG (Zhongshanjinqiao, PV6001) for 30 min. After washing in PBS, diaminobenzedine (DAB) substrate was added and the sections were counterstained with Mayer's hematoxylin. The sections were mounted and examined. Negative control slides were processed through the same steps with omission of the primary antibody. Image analysis of Kindlin-2 immunostaining was detected within the AOI and quantified using ImagePro Plus 6.0 (Media Cybernetics Inc, Bethesda, MD).

### Apoptosis Assay and Immunofluorescent Staining

TUNEL (terminal deoxynucleotidyl transferase dUTP nick-end labeling) BrightRed Apoptosis Detection Kit (Vazyme, A113) was used to detect the cell apoptosis according to the manufacturer's instructions. In belief, the embryos and adult tissues sections were hydrated and incubated with protein Kinase (20 μg/ml) at room temperature for 20 min, washed with PBS three times and then incubated with equilibration buffer for 10 min, then TdT (terminal deoxynucleotidyl transferase) reaction mixture was added to the tissues for 1 h incubation at 37°C, washed with PBS three times, incubated with BSA (5 mg/ml) for stopping the reaction if necessary. Sections were counterstained with DAPI for 5 min. The data were obtained using a confocal microscope (Meta Zeiss LSM 780, objective W.I. 63X). If other antibodies were needed to co-incubated at the same section, the sections were blocked by 3% H_2_O_2_, antigen-retrieval as described before, probed with antibody overnight and then incubated with fluorescence-labeled IgG, all these were operated before protein Kinase incubation.

### Intracellular Ca^2+^ Measurement

Mouse PVSMC were loaded with 4 mM Fluo3-AM to detect the change of intracellular Ca2+ under an inverted confocal laser scanning microscopy (Meta Zeiss LSM 780, objective W.I. 63X). Cells cultured in cover glass-bottom dishes were incubated with 4 mM Fluo3-AM and 0.02% Pluronic F-127 for 30 minutes, and rinsed 3 times with PBS. Confocal microscopy with excitation and emission wavelengths of 506 nm and 526 nm, respectively, was used to detect fluorescence intensity. By sketching the contour of the whole cell, the fluorescence signal and image was captured every 10 seconds, and this procedure continued for 400 seconds. At about 60 seconds, 100 mM Angiotensin (sigma A9525) was immediately injected into the dishes with a pipettor. The signal of at least 20 different cells in the dishes was acquired each time. The fluorescence signal was analyzed by ImagePro Plus 6.0. F0 was the basal fluorescence signal, and Fe was the experimental signals. The equation Fe /F0 was used to express the relative level of intracellular calcium.

### Whole-gut Transit Time Test

To determine function of gastrointestinal emptying, a whole-gut transit time test was performed as described before 7. 100 μl charcoal test meal (5% [w/v] charcoal in 0.9% NaCl) was injected by orogastric gavage and monitored feces for the first appearance of black dye.

### Aortic Ring Contraction and Relaxation Measurement

The multiwire myograph system (DMT 620M) was used for mounting of descending aortas ring and measurement of mechanical force, the system connects for vacuum and gassing, as well as heating are provided in the myograph interface, allowing for all chambers to be smoothly maintained under physiological settings (37 °C, and bubbled with 21% O_2_: 5% CO_2_). The myograph system was connected to a PC via an amplifier (Power Lab 8/35, AD Instruments) for continuous measurement of isometric tension using data acquisition software (Lab Chart Pro Version 8.0).

The mice were killed by cervical dislocation. The aortas (1.5 mm long) or segments (6 mm long) of the jejunum and ileum were placed in the Krebs solution (KCl 4.7 mmol/L, MgSO_4_ 0.57 mmol/L, KH_2_PO_4_ 1.16 mmol/L, CaCl_2_ 2.5 mmol/L, NaHCO_3_ 24 mmol/L, Glucose10 mmol/L and NaCl 118 mmol/L, pH 7.4). Aortic rings were precontracted with KCL (60mM) to maximal contraction (Emax) first, and then washed and stabilized. Phenylephrine (Phe, Sigma, P124000) cumulative concentration from 3 nM to 10 mΜ was performed to measure Phe-induced contration, and washed with Krebs solution four times. Second, aortic rings were precontracted with Phe (1μM), when the maximal contraction reached a plateau, cumulative concentration-response curves (from 3 nM to 10 μM) for Acetylcholine chloride (Ach, Sigma, A2661) were generated. After the completion of the curves, the rings were washed and stabilized. Third, the aortic rings were incubated with 100 μM NG-nitro-L-arginine (L-NAME) for 30 min, an inhibitor of NOS. Phe cumulative concentration from 3 nM to 10 μM and sodium nitroprusside (SNP, Sigma, PHR1423) cumulative concentration from 1nM to 10 μM was performed to measure endothelial independent contraction and relaxation. ΔE/Emax was count to express the relative level of contraction.

### Transmission Electron Microscopy

The descending aortas and colons were harvested from Kindlin-2^fl/fl^ MYH-cre- and Kindlin-2^fl/fl^ MYH-cre+ mice 5 weeks after Tamoxifen treatment, and cut into 1mm^3^ transverse and longitudinal ultrathin sections, and the sections were fixed in 2.5% glutaraldehyde at room temperature for 4 h. Then the samples were post-fixed with 2% osmium tetroxide, block-stained with 2% uranyl acetate, dehydrated in an acetone series, and then embedded in Epon 812. Using semi-thin sections stained with Toluidine Blue as reference, the sections were cut into ultra-thin sections and observed under a transmission electron microscope (JEM-1400, JEOL Ltd., Tokyo, Japan).

### RNA Isolation and qRT-PCR Analysis

Total RNA was isolated from primary SMC using Trizol reagent (Invitrogen).

The first chain cDNA was synthesized using HiScript Q Select RT SuperMix for qPCR (+gDNA wiper, Vazyme, China), RT-PCR analyses were performed on LightCycler 96 detection system (Roche) according to the manufacturer's instructions. All mRNA expression levels were normalized to signal of GAPDH gene. Sequences of corresponding primers were designed as follows:

FERMT2, forward, GGGGTGATGCTGAAGTTGGT, reverse, CTGCCTGGATGCCACACTTA. MYH11, forward, TCCGTGCTACACAACCTGAG, reverse, TGTCTGCGATGGCGTAGATG. ACTA2, forward, CCAAGCACTGTCAGGAAT, reverse, AGAGGAACCTAATCTGTGTC. STIM1, forward, TTCTTTGGGGGCTCCTTCTG, reverse, GCACAGGGGCTTGTCAATTC. S100A14, forward, CTCTGTGGCGGGTAAAAAGG, reverse, TTGCCCAGGTTGGCAATTTTC.

### Statistical Analysis

All the results are expressed as the mean±SEM. Statistical analysis was performed using GraphPad Prism 5.0 software. For statistical comparisons, we first evaluated whether the data were normally distributed. Then, the Brown-Forsythe test was used to check for similar variances among normally distributed data; if the variances were similar, we applied Student *t* test for 2-group comparisons. Nonparametric tests were used where data were not normally distributed. In all cases, statistical significance was concluded where the 2-tailed probability was <0.05. The details of the statistical analysis applied in each experiment are presented in the corresponding figure legends.

## Results

### Smooth muscle-specific knockout of the Kindlin-2 gene resulted in embryonic lethality

**Figure 1:**
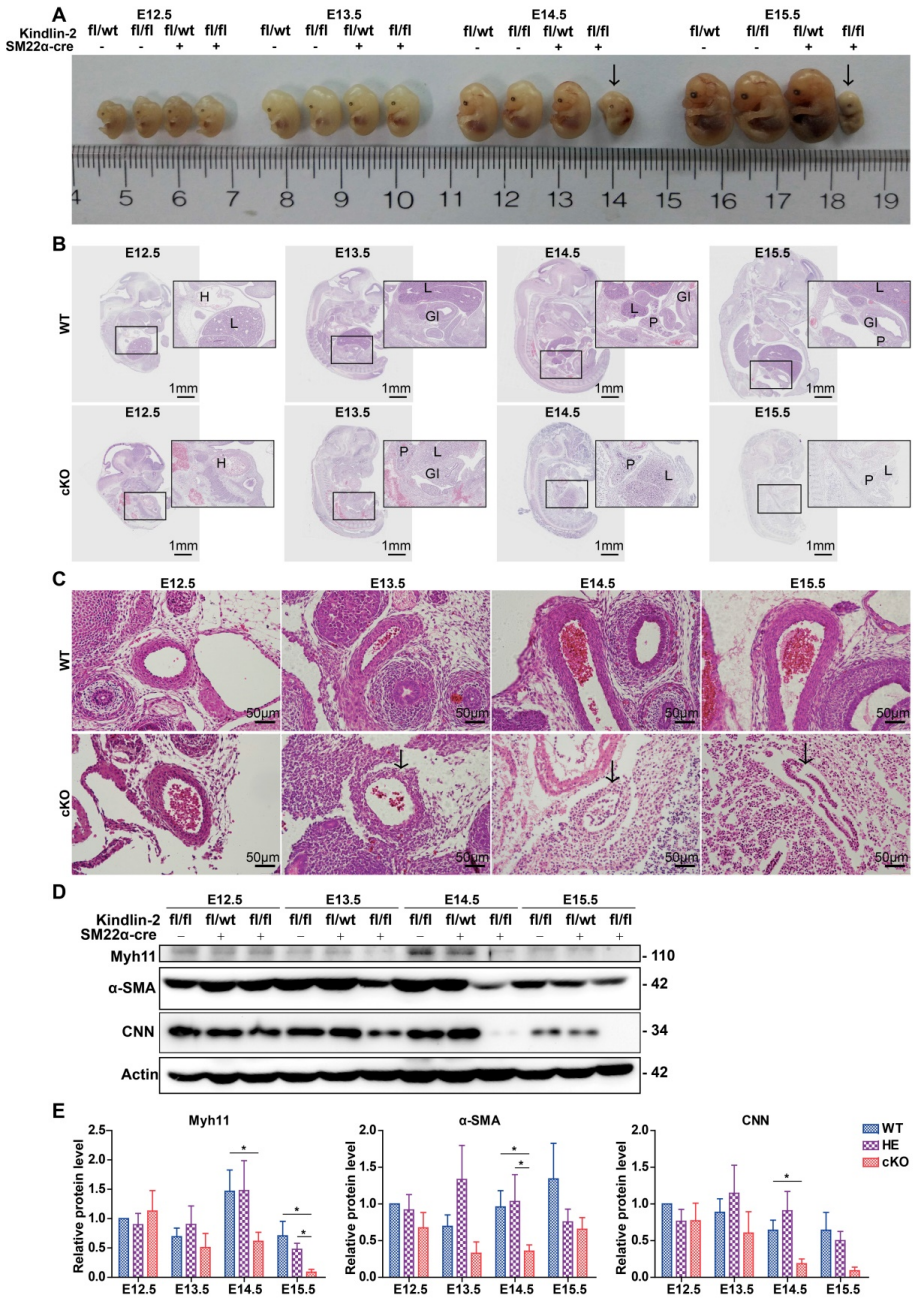
** The injury of smooth muscle caused by specific knockout of Kindlin-2 leads to embryonic lethality.** (A) Gross appearance of E12.5-15.5 embryos with different genotype. Embryos were fixed with paraformaldehyde. cKO (Kindlin-2^fl/fl^;SM22α-cre+) embryos are lethal at E14.5 (arrows). (B) Whole embryo sections were stained with hematoxylin and eosin (HE). Representative images and morphological abnormality are presented. The WT (Kindlin-2^fl/fl^;SM22α-cre-, Up panel) and cKO mice (Kindlin-2^fl/fl^;SM22α-cre+,Lower panel) were presented. H: heart; L: liver; GI: gastrointestinal tract; P: pancreas. Scale bar, 1mm. (C) HE staining of embryonic arteries from E12.5-E15.5. Scale bar, 50μm (D) Western blot analysis of whole embryo lysis from E12.4-E15.5, test for Myh11, α-SMA, CNN expression levels respectively. (E) Quantitation of (D) and two other independent experiments. Data were presented as the mean ± SEM. *p < 0.05 by student's t-test. See Figure S1, S2, Table S1, S2 and S3 for additional information.

Kindlin-2 is known to be expressed preferentially in the heart, skeleton, myocardium, and smooth muscles 33. However, the function of Kindlin-2 in smooth muscles remains elusive. To clarify the role of Kindlin-2 in smooth muscle cells, we established a mouse line with Kindlin-2 knocked out specifically in smooth muscle cells. Kindlin-2^fl/fl^ mice (Figure S1A) were crossed with SM22α-Cre transgenic mice to generate Kindlin-2^fl/wt^;SM22α-cre+ mice 31, 34, which were further crossed with Kindlin-2^fl/fl^ mice to obtain Kindlin-2^fl/fl^;SM22α-cre+ mice (Figure S1B). Littermate Kindlin-2^fl/fl^;SM22α-cre-, Kindlin-2^wt/wt^;SM22α-cre+, and Kindlin-2^wt/wt^ mice were used as non-depletion controls. Different genotypes were distinguished by polymerase chain reaction (PCR) amplification and DNA agarose gel electrophoresis with specific primers (Figure S1C). Among 134 newborn offspring, 50 (37.3%) were Kindlin-2^fl/wt^;SM22α-cre- mice, 43 (32.1%) were Kindlin-2^fl/fl^;SM22α-cre- mice, and 41 (30.60%) were Kindlin-2^fl/wt^;SM22α-cre+ mice. However, genotyping showed that no Kindlin-2^fl/fl^;SM22α-cre+ (referred to as cKO hereafter) offspring were born. The birth ratio of the genotype did not conform to Mendelian inheritance (Table S1). At E14.5-E15.5, 77.77% of the embryos were normally developed (presumptive wild and heterozygous types), and 22.22% were severely misshapen or already resorbed (Figure S1D and Table S2). Subsequent PCR-genotyping analysis revealed that this 22.22 % of embryos were all cKO embryos, which suggested that specific depletion of Kindlin-2 in SMCs was embryonic lethal. To determine when the cKO embryos died, we used a time-mating strategy to harvest embryos at different developmental stages. cKO embryos at E14.5 displayed a pale gross appearance, multi-organic hematomas, and bloody amniotic fluid (data not shown), whereas the wild and heterozygous littermates were normal, indicating that embryonic death occurred at E14.5. Immunohistochemical staining revealed that Kindlin-2 protein expression had decreased significantly in the smooth muscle from E12.5 cKO embryos, compared to those of the Kindlin-2^fl/fl^;SM22α-cre- mice (referred to as WT mice hereafter; Figure S1E), suggesting that the knockout mouse model was effective. Vasculogenesis occurs in early developing embryos, enabling the establishment of the vascular system 35. Kindlin-2 is expressed in both the epithelium and smooth muscle 36, and Kindlin-2 is reported to facilitate angiogenesis as heterozygous mice showed significantly reduced angiogenesis in growing tumors and Matrigel implants 29. In this study, we also generated vascular endothelium-specific Kindlin-2-knockout mice using the Tie2-cre system, but no Kindlin-2^fl/fl^;Tie2-cre+ (homozygous-depletion genotype) offspring were born (Table S3) and no Kindlin-2^fl/fl^;Tie2-cre+ embryos were found at E10.5 uteruses. It is already known that SMC growth and recruitment occurs somewhere around E11.5-E12.5 in mice 37. Smooth muscle alignment is thought to occur at about E13.5 37. To identify morphological differences between smooth muscles from cKO and control embryos, cKO embryos were examined at E13.5. Few abnormalities were noted in terms of gross appearance, and no marked differences were found in terms of the overall body size (Figure 1A). However, hematoxylin and eosin (H&E) staining revealed that cKO mutants exhibited multi-organ defects at this time point (Figure 1B). At E14.5-E15.5, the cKO embryos were dead or resorbed, and the organs had become vague and difficult to stain. During embryogenesis, SMC recruitment from their different progenitors (for instance, somites or splanchnic mesoderm origins) is important for the formation of the embryonic vascular system 1, and we found that cKO vessels showed impaired structures, thinner vessel walls, and smaller diameters (Figure 1C). However, the control internal organs developed normal structures and exhibited moderate growth over time. At E12.5, the heart, lungs, and liver had begun to grow, and cKO embryos showed little difference from WT embryos but developed hematomas (Figure 1B). Notably, the Kindlin-2^fl/wt^;SM22α-cre+ were viable and fertile and did not show any marked abnormalities (Figure S2). Therefore, we spared the heterozygous group and used the WT littermates as the only control group for the rest of the study. Western blot analysis of whole embryo lysates showed that, in the cKO embryos, the expression levels of smooth muscle makers (Myh11, α-SMA, and CNN) decreased at E13.5 or E14.5, whereas the WT embryos exhibited normal expression levels during embryonic development (Figure 1D-E). SMC differentiation was induced at E9.0-9.5 37 as the embryos grow smooth muscle decreases in overall proportion. These findings explain why all 3 smooth muscle markers were partially downregulated at E15.5 (Figure 1D and Figure S2). Taken together, these results indicate that the depletion of Kindlin-2 causes severe embryonic abnormality at E13.5 and embryonic death at E14.5, with concomitant downregulation of smooth muscle marker proteins. Therefore, Kindlin-2 serves an essential role in embryonic development by affecting smooth muscle.

### Kindlin-2 depletion impaired smooth muscle development by promoting apoptosis in mouse embryos

**Figure 2:**
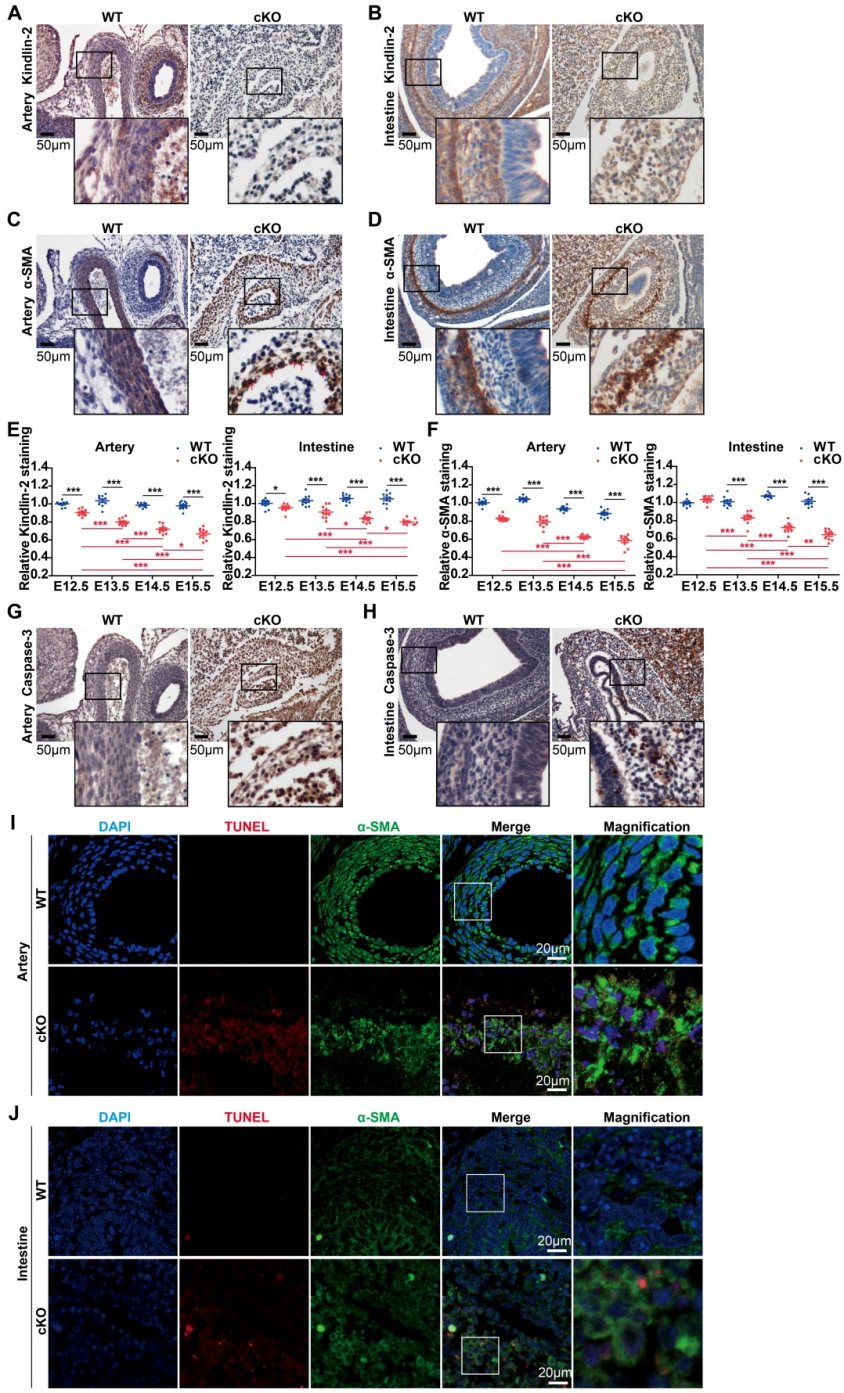
** Specific knockout of Kindlin-2 leads to apoptosis in vascular and intestinal smooth muscles.** (A) Immunohistochemical staining of E14.5 WT and Kindlin-2 cKO embryonic cross-sections for Kindlin-2 in arteries. Scale bar, 50μm. (B) Immunohistochemical staining of E13.5 WT and Kindlin-2 cKO embryonic sections for Kindlin-2 in intestine. Scale bar, 50μm. (C) Immunohistochemical staining of E14.5 WT and Kindlin-2 cKO embryonic cross-sections for α-SMA in arteries. Red arrows point out karyopyknosis. Scale bar, 50μm. (D) Immunohistochemical staining of E13.5 WT and Kindlin-2 cKO embryonic sections for α-SMA in intestine. Scale bar, 50μm. (E) Quantitation of Kindlin-2 expression of **Figure S3A and C** by Image J, data are mean ± SEM. *p < 0.05, **p < 0.01 and ***p < 0.001 by student's *t*-test. (F) Quantitation of α-SMA expression of **Figure S3B and D** by Image J, data are mean ± SEM. *p < 0.05, **p < 0.01 and ***p < 0.001 by student's *t*-test. (G) Immunohistochemical staining of E14.5 WT and Kindlin-2 cKO embryonic cross-sections for cleaved caspase-3 in arteries. Higher magnified views of highlighted regions were shown below. Scale bar, 50μm. increased protein expression levels of caspase-3 shows apoptosis of cKO mice compared with WT littermate. (H) Immunohistochemical staining of E13.5 WT and Kindlin-2 cKO embryonic sections for cleaved caspase-3 in intestine. Higher magnified views of highlighted regions were shown below. Scale bar, 50μm. (I) Representative immunofluorescence photographs of arteries from E14.5 WT and Kindlin-2 cKO embryonic cross-sections. Sections were co-stained for α-SMA and TUNEL. Higher magnified views of highlighted regions were shown on the right. Scale bar, 20μm. (J) Representative immunofluorescence photographs of intestines from E13.5 WT and Kindlin-2 cKO embryonic sections. Sections were co-stained for α-SMA and TUNEL. Scale bar, 20μm. Higher magnified views of highlighted regions were shown on the right. See Figure S3 for additional information.

Given that cKO embryos showed marked abnormalities during development (Figure 1B-C), we focused on organs with abundant smooth muscles, such as arteries and intestines. Immunohistochemical staining revealed that Kindlin-2 was nearly depleted in cKO arteries at E14.5 when compared to the WT littermates (Figure 2A). At E13.5, Kindlin-2 was partially depleted in cKO intestines (Figure 2B). cKO embryos were less well developed and showed incoherent smooth muscles, as indicated by α-SMA staining in the tunica media. The number of vascular smooth muscle layers markedly decreased from 6 or 7 layers to no more than 3 layers in the same-sized arteries of cKO embryos (Figure 2C). Compared with the wild type mice, the smooth muscle of the gastrointestinal tract in the cKO group became thinner or disappeared (Figure 2D). The quantitation of Kindlin-2 levels in the arteries and intestines of embryos (Figure 2E) showed that Kindlin-2 expression greatly decreased with embryonic development from E13.5 to E15.5 in cKO mice and with the decrease of Kindlin-2, the number of smooth muscle cells continued to decline significantly (Figure S3 and Figure 2F). Taken together, these results suggest that Kindlin-2 depletion leads to dysgenesis of smooth muscle layers in mouse embryos.

Given that Kindlin-2 is required for the maintenance of cell viability 38, we, therefore, sought to answer why smooth muscle layers in mouse embryos are poorly developed with Kindlin-2 depletion. We performed terminal deoxynucleotidyl transferase dUTP nick-end labeling (TUNEL) assays to measure apoptosis when Kindlin-2 was depleted. The results showed increased levels of cleaved (activated) caspase-3 in cKO mice compared to the WT littermates (Figure 2G-H). To further characterize the SMCs, we co-stained aortic and intestinal tissues for TUNEL assays and α-SMA detection. We found that DNA fragmentation increased in α-SMA-positive SMCs in both the arteries and intestines of cKO mice (Figure 2I-J), indicating that apoptosis increased in the SMC-rich medial layers. Collectively, these data suggest that Kindlin-2 depletion in smooth muscles promotes apoptosis and jeopardizes smooth muscle-layer formation.

### Depletion of Kindlin-2 in smooth muscles led to intestinal obstruction and adult mouse death

The aforementioned findings demonstrated that smooth muscle-specific Kindlin-2 depletion was embryonic lethal and that Kindlin-2 is essential for smooth muscle development during embryogenesis. To uncover the role of Kindlin-2 in adult smooth muscles, we established a mouse line where Kindlin-2 could be specifically knocked out in smooth muscles in an inducible manner (referred to as iKO mice hereafter). MYH-cre transgenic mice were used to generate adult mice capable of Kindlin-2-specific knockout in SMCs, based on MYH11 gene promoter-driven Cre expression that was induced by tamoxifen treatment 39. Because the Cre excision was permanent, this model provided permanent Kindlin-2 depletion in virtually all mature (MYH11+) SMCs upon tamoxifen injection. This, therefore, ensured that Kindlin-2 was depleted specifically in adult smooth muscles, irrespective of continued expression of Myh11, SM22α, or other SMC marker genes. The cross-breeding routine was similar to that used to generate the cKO mice, which is described in detail in Figure S4.

**Figure 3:**
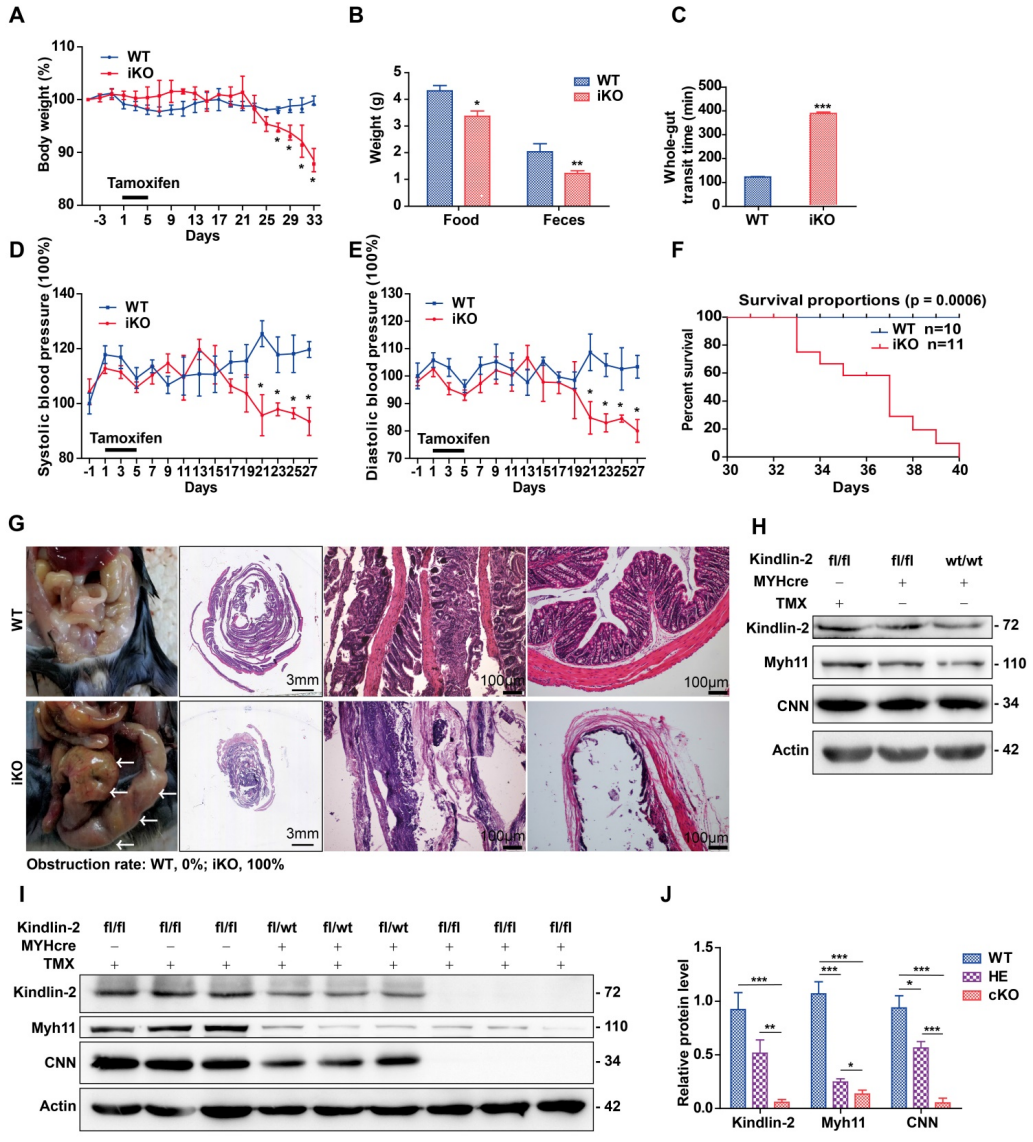
** Specific knockout of Kindlin-2 in adult smooth muscle causes individual death from intestinal obstruction.** (A) A body-weight based growth curve for Kindlin-2^fl/fl^;MYH-cre-(WT) and Kindlin-2^fl/fl^;MYH-cre+ mice 39 after tamoxifen injection (n=4), *p < 0.05, **p < 0.01, ***p < 0.001. (B) Food intake and feces excretion after 4 weeks of tamoxifen injection (n=4 each group), *p < 0.05, **p < 0.01. (C) Whole-gut transit time for WT and adult iKO mice (n=4), ***p < 0.001. (D) Systolic blood pressure curve for WT and iKO mice, tamoxifen injection was treated at day 1, n=4, *p < 0.05 (E) Diastolic blood pressure curve for WT and iKO mice, tamoxifen injection was treated at day 1, n=4, *p < 0.05. (F) Kaplan-Meier survival plot of WT (n=11) versus iKO littermates (n=10). Mice were treated with tamoxifen at 6-8 weeks, and the plot begins 1 day later. All iKO mice developed huge colon-like obstruction and died before 40 days, while the control mice grown normally. *p* = 0.0006. (G) Left, ventral view of colons on WT and iKOmice at the time of necropsy, 5 weeks after tamoxifen treatment, iKO mice displayed a huge colon-like phenotype (white arrows). Middle, intestinal swiss roll after HE staining. Scale bar, 3mm (the second column), 100μm (the third and fourth columns). (H) Western blot analysis of Kindlin-2, smooth muscle markers Myh11, CNN and Actin protein expression of colon in three types of control mice, Kindlin-2^fl/fl^;MYH-cre-, Kindlin-2^wt/wt^; MYH-cre+ mice underwent tamoxifen injections, and Kindlin-2^fl/fl^;MYH-cre+ with corn oil injections, colons were harvested after 4 weeks of rest period, no significant difference was found. (I) Western blot analysis of Kindlin-2, smooth muscle markers Myh11, CNN and Actin protein expression of colon in WT, Kindlin-2^fl/wt^;MYH-cre+, and iKO mice. 4 weeks after tamoxifen treatment. (J) Quantitation of Fig I, *p < 0.05, **p < 0.01, ***p < 0.001. See Figure S4 for additional information.

When performing experiments, we used iKO mice and their sibling age-matched Kindlin-2^fl/fl^;MYH-cre- controls (referred to as WT hereafter). These adult mice were injected with tamoxifen once per day for 5 consecutive days. From day 27 after the tamoxifen injections, the iKO mice showed significantly reduced body weights, compared to the controls (Figure 3A). At day 33, the weight of iKO mice was 33.1% less than that of the WT controls (19.2 ± 3.35 g vs. 28.7 ± 0.3 g, n = 4; *p* < 0.001). Three weeks after tamoxifen injection, the iKO mice showed reduced physical body motility (data not shown), and food intake and feces excretion were markedly reduced (Figure 3B). The motility of the intestines was determined by performing functional assays. Charcoal, a black dye, was injected into the stomach, and the stool was monitored. The stool whole-gut transit time in iKO mice was about 3 times longer than that of WT mice (Figure 3C). We also monitored the blood pressure of mice using a CODA non-invasive blood pressure monitoring system 40. At 3 weeks after tamoxifen injection, both the systolic and diastolic blood pressure of iKO mice had decreased severely (Figure 3D-E), indicating that the vascular smooth muscle tone had decreased. However, the heart and other organs of iKO mice did not show significant changes, e.g., hemorrhaging and bloody exudate (data not shown). At day 33 after the tamoxifen injections, the iKO mice began to die, and all iKO mice (n = 11) died before day 40. However, all WT mice survived normally (n = 10; Figure 3F). Autopsies showed that the iKO mice developed intestinal obstructions, and the incidence of colon expansion was 100% (Figure 3G). Western blot analysis revealed that none of the three different controls showed significantly different expression of Kindlin-2 or smooth muscle markers (Figure 3H). However, the Kindlin-2 level dramatically decreased in Kindlin-2^fl/wt^; MYH-cre+ and iKO colons, respectively, as did the smooth muscle markers Myh11 and CNN (quantification is shown in Figure 3I-J). No death or obvious phenotypic changes occurred in heterozygous mice (data not shown). Taken together, these data indicated that depleting Kindlin-2 in adult SMCs led to the loss of smooth muscle markers, incomplete intestinal motility, and death due to obstructive enteropathy.

### Kindlin-2 loss disrupted smooth muscle structures and caused apoptosis in adult mice

**Figure 4:**
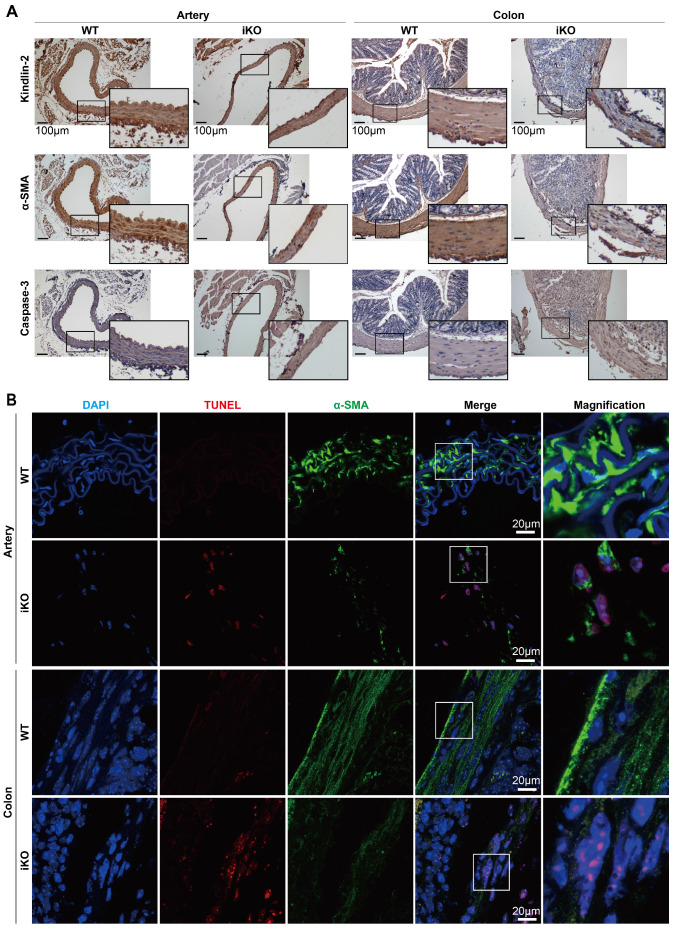
** Depletion of Kindlin-2 in adult smooth muscle leads to apoptosis.** (A) Kindlin-2, α-SMA, cleaved caspase-3 immunohistochemical staining of aortic cross-sections (left) and colon (right). WT and iKO mice were treated with tamoxifen injection parallelly, ascending aortas and sigmoid colons were excised and fixed 14 days after injection. Scale bar, 100μm. (B) Immunofluorescence of TUNEL (red), DAPI (blue) in combination with α-SMA (green) on cross-sections of ascending aortas (up) and sigmoid colons (below). Higher magnified views of highlighted regions were shown on the right. Scale bar, 20µm. See Figure S5 for additional information.

Given that the Kindlin-2 loss caused death at E14.5 in embryonic mice and led to intestinal obstruction in adult mice, we next studied why Kindlin-2 is essential for smooth muscle formation and functional execution. To this end, we examined the role of Kindlin-2 in maintaining smooth muscle tissue structure. After 4 weeks of tamoxifen treatment, we harvested arteries and colons (which are rich in smooth muscle tissue) from the treated mice. Immunohistochemical staining (IHC) was then performed to evaluate the changes in Kindlin-2, α-SMA, and caspase-3 expression (with or without Kindlin-2) in smooth muscles. The IHC results showed that complete Kindlin-2 knockout caused the smooth muscle layers to become thin and acquire a disordered arrangement, both in arteries and colons in iKO mice, whereas expression of the smooth muscle marker α-SMA decreased greatly, and that of the apoptosis marker caspase-3 increased significantly (Figure 4A). Similarly, TUNEL experiments also showed that apoptosis increased greatly in smooth muscles from iKO mice compared to those from WT mice (Figure 4B). In contrast, the heterozygous knockout of Kindlin-2 in adult mice did not show observable morphological changes, when compared with WT mice (Figure S5). Together, these findings support the conclusion that depleting Kindlin-2 in adult and embryonic smooth muscle showed similar molecular phenotypes and disrupted cellular structures.

### The loss of Kindlin-2 destroyed the ultrastructure of adult smooth muscles

**Figure 5:**
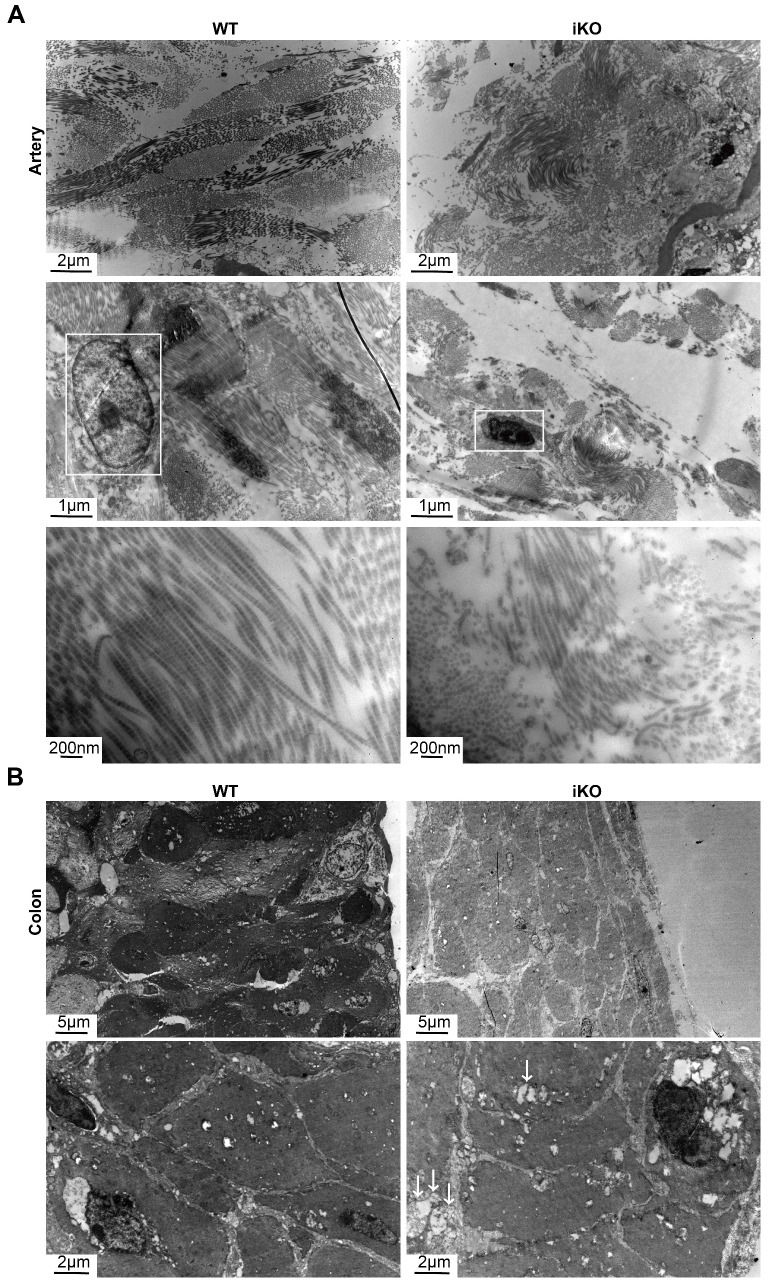
** iKO mice exhibited the ultrastructure impairment of SMC.** (A) Transmission electron microscope (TEM) images of aortic smooth muscle. WT and iKO mice were treated with tamoxifen injection parallelly, and ascending aortas were excised and fixed 14 days after injection. White boxes point out nuclei, showing karyopyknosis in iKO SMC. Length of longitudinal smooth muscle filaments, WT group:13.96±3.22μm (up left), iKO group: 2.71±0.57μm (up right).* p* < 0.001; Upper panel: scale bar, 2μm, middle scale bar, 1μm below scale bar, 200nm. (B) Transmission electron microscope (TEM) images of colonic smooth muscle. WT and iKO mice were treated with tamoxifen injection parallelly, and sigmoid colons were excised and fixed 14 days after injection. White arrows: vacuoles. Diametre of smooth muscle, 52.76±0.95μm (up left: WT group) vs. 37.48±2.56μm (up right: iKO group). *p* < 0.01; Upper panel: scale bar, 5μm, below scale bar, 1μm.

Given that the loss of Kindlin-2 drastically changed the morphology of smooth muscles in adult mice, we next examined the ultrastructural changes that occurred with Kindlin-2 depletion. To this end, we studied aortic and colonic smooth muscles after Kindlin-2 depletion by electron microscopy. Our results demonstrated that the longitudinal myofibrils of iKO smooth muscles became shorter than those of the WT mice (2.71 ± 0.57 μm vs. 13.96 ± 3.22 μm, *p* < 0.01; Figure 5A). Moreover, the myofibrils become curly and irregular in iKO vascular walls (Figure 5A), indicating that almost all myofibrils were broken without Kindlin-2. Further, the diameter of intestinal smooth muscle fibers decreased significantly (iKO mice, 37.48 ± 2.56 μm; WT mice, 52.76 ± 0.95 μm; *p*<0.01; Figure 5B). Decreased electron density and increased vacuoles in iKO intestinal SMCs suggested that the number of myofibrils and/or cell organelles decreased greatly compared with WT smooth muscles. These results indicate that Kindlin-2 deficiency decreased the number of SMCs and changed the myofilament structure in smooth muscles. Thus, the observed ultrastructural changes were in accordance with the decline of smooth muscle markers identified in the aforementioned findings. In addition, Kindlin-2 depletion also led to the concentration of nuclear chromatin and the disappearance of the nuclear membrane.

### Kindlin-2 depletion jeopardized the contractile function of smooth muscles

**Figure 6:**
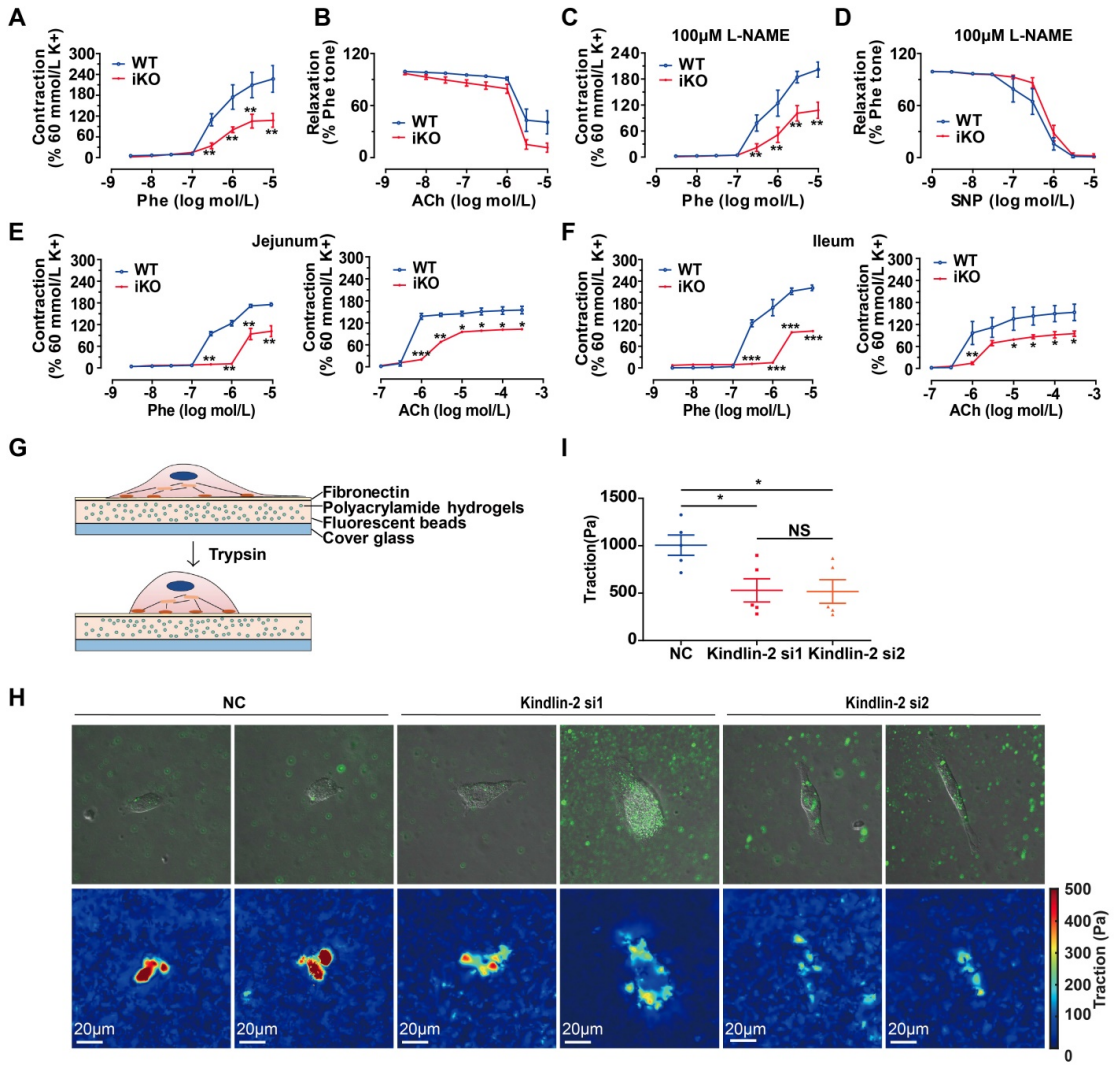
** Loss of Kindlin-2 damaged the contractile function of adult smooth muscle.** (A) Cumulative concentration-response curves (from 3nM to 10 μM) for the phenylephrine- (Phe-) induced contraction of aortic rings from WT and KO mice. Mice were treated with tamoxifen injection at 8 weeks and rested for 2 weeks before the experiment. Data were presented as the mean ± SEM (n=6). **p < 0.01 by student's t-test. (B) Cumulative concentration-response curves (from 3nM to 10 μM) for the acetylcholine-(ACh-) induced relaxation of aortic rings from WT and iKO mice. Data were presented as the mean ± SEM (n=6). (C) Cumulative concentration-response curves (from 3nM to 10 μM) for the Phe-induced contraction of aortic rings from WT and iKO mice. Rings were incubated with 100μM L-NAME before Phe-induced contraction. Data were presented as the mean ± SEM (n=6). **p < 0.01 by student's t-test. (D) Cumulative concentration-response curves (from 3nM to 10 μM) for the SNP-induced relaxation of aortic rings from WT and iKO mice. Rings were incubated with 100μM L-NAME before Phe-induced contraction. Data were presented as the mean ± SEM (n=6). (E) Cumulative concentration-response curves (Phe: from 3nM to 10 Μm, Ach: from 100nM to 300 μM) for the Phe- and ACh -induced contraction of jejunum from WT and iKO mice. Data were presented as the mean ± SEM (n=4). *p < 0.05, **p < 0.01 and ***p < 0.001 by student's t-test. (F) Cumulative concentration-response curves (Phe: from 3nM to 10 Μm, Ach: from 100nM to 300 μM) for the Phe- and ACh -induced contraction of ileum from WT and iKO mice. Data were presented as the mean ± SEM (n=4). *p < 0.05, **p < 0.01 and ***p < 0.001 by student's t-test. (G) Schematic illustration of a cell traction force microscopy (CTFM) experiment depicts elastic substrate deformed by an adherent cell. The cell is detached with trypsin to monitor displacement field. (H) Traction forces of PVSMC using CTFM. The traction force map is color coded, with red representing the highest force and blue representing the lowest. (I) Quantitation of the highest traction force in (H). Data were presented as the mean ± SEM (n=5). *p < 0.05 by student's t-test.

The major functions of SMCs are contraction and relaxation. Vascular smooth muscles regulate the blood pressure and flow through, whereas gastrointestinal SMCs regulate peristalsis and defecation. SMC contractile force requires cyclic interactions between the smooth muscle proteins, α-SMA, and Myh11 13. To determine the contractile properties of Kindlin-2-deficient smooth muscles, we measured the tension in isolated aortic rings or intestinal rings of iKO and WT mice (after 2 weeks of tamoxifen injections) with a myograph, following a previously described protocol 41, 42. Compared to those from WT mice, iKO aortas exhibited a less sensitive response to phenylephrine (Phe) in terms of endothelium-dependent contraction (Figure 6A), but relaxation was fully promoted by the muscarinic agonist, acetylcholine (ACh), which reverses contraction stimulated by Phe (1 μM; Figure 6B). Endothelium-dependent vasodilation is mainly determined by the activity of endothelial nitric oxide synthase (eNOS), whereas ACh is a vasodilator that relaxes vessels mainly by activating eNOS, thereby increasing NO production 43, 44. To measure endothelial-independent contraction and relaxation, the aortic rings were incubated with 100 μM NG-nitro-L-arginine (L-NAME), an eNOS inhibitor used to block endothelium-dependent vasodilation before incubation with Phe and sodium nitroprusside (SNP).

The results showed that iKO aortas also exhibited a less sensitive response to Phe, indicating that the impaired contraction was derived from smooth muscles (Figure 6C). Relaxation was also fully reversed with SNP (Figure 6D). Jejunal rings from iKO mice showed a less sensitive response to Phe (3 μM vs. 300 nM, *p*<0.01) and Ach (3 μM vs. 1 μM, *p*<0.001) (Figure 6E). Similar results were observed with ileal tissues in response to Phe (3 μM vs. 300 nM, *p*<0.001) and Ach (3 μM vs. 1 μM, *p*<0.01) (Figure 6F). Together, these data indicated that the loss of Kindlin-2 could seriously damage the contractile function of both vascular and gastrointestinal smooth muscles. The loss of Kindlin-2 in smooth muscles decreased the reactivity to Phe, ACh, and SNP. However, Kindlin-2 depletion did not significantly affect the relaxation of vascular smooth muscles, which may be due to Kindlin-2 depletion is specific in smooth muscle rather than in endothelium. Meanwhile, we could not rule out the possibility that the measurement of diastolic function is limited when the systolic function of vascular smooth muscles has been severely damaged either. SMCs are anchorage-dependent cells that must balance the contraction against adequate adhesion to the substrate. Therefore, the forces between SMCs and the cell-adhering substrate can indirectly determine SMC contractility 45. Thus, cell traction force microscopy (CTFM) was used to determine the traction forces of SMCs. Briefly, CTFM was used to measure cell contraction-dependent displacement of fluorescent beads embedded in polyacrylamide hydrogels (Figure 6G), and then the cell-traction forces were computed. Using time-lapse microscopy 46 and CTFM, the traction forces of primary vascular smooth muscle cells (PVSMCs) at different time points were determined, revealing that the overall traction forces of PVSMCs markedly decreased upon Kindlin-2 knockdown by RNAi (Figure 6H-I). Taken together, these data demonstrated that Kindlin-2 played an important role in controlling smooth muscle contraction.

### Kindlin-2 depletion downregulated the expression of key calcium-regulating proteins in smooth muscles

**Figure 7:**
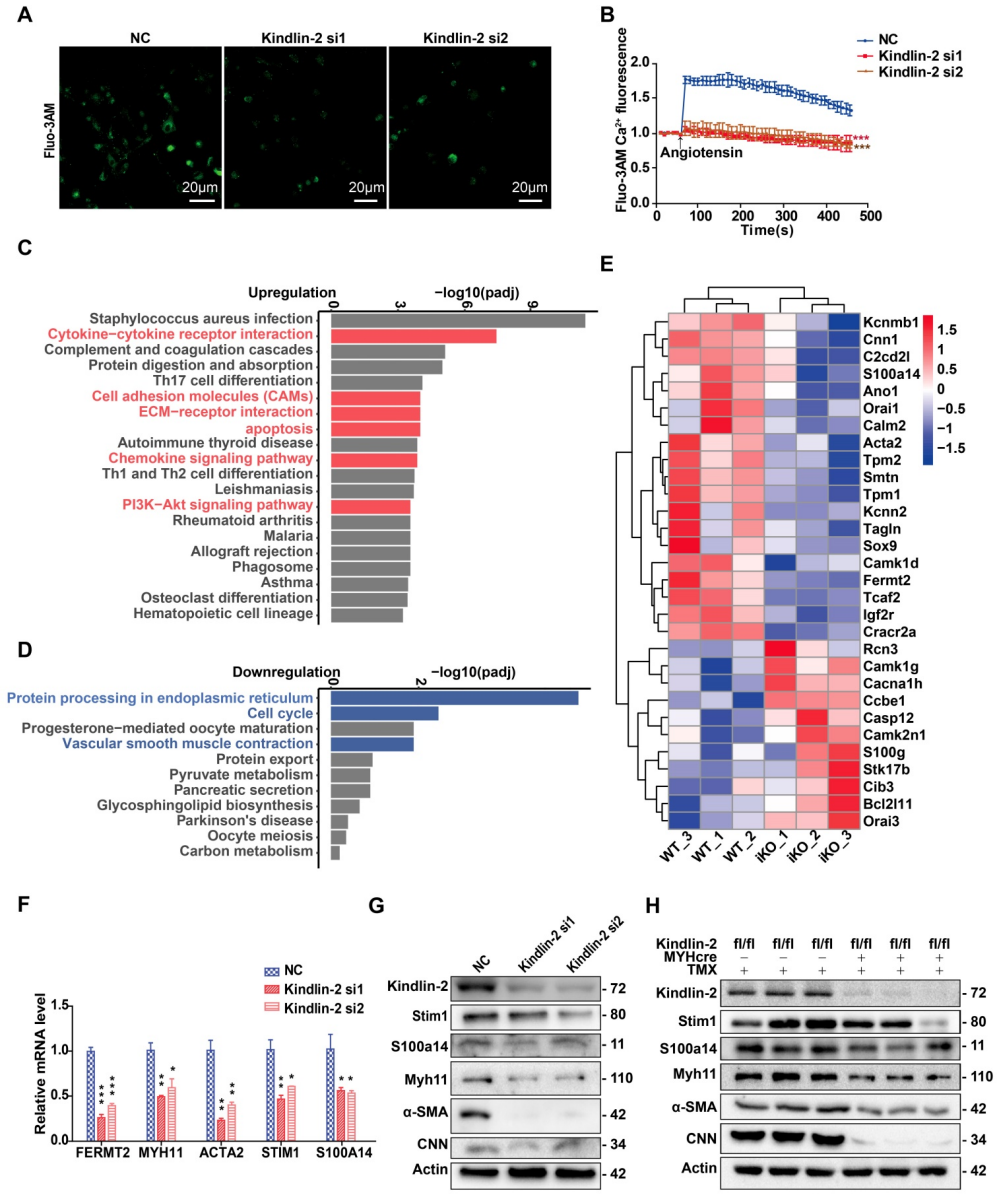
** Depletion of Kindlin-2 downregulates calcium binding proteins and SOCE signaling.** (A) Cells preloaded with fluo3-AM were stimulated by angiotensin and examined under a confocal microscope. Serial fluorescent images were captured every 20 seconds. Some representative images (taken at 50s after stimulation) are shown. Scale bar, 20μm. (B) Relative fluo3-AM fluorescence values were calculated as ΔF/F0 to represent the relative Ca^2+^ levels. (C) KEGG analysis of 20 most upregulated pathways of identified genes in iKO group than WT controls. (D) KEGG analysis of 11 most downregulated pathways of identified genes in iKO group than WT controls. (E) A heatmap of identified genes (>2 fold change) related to KEGG Smooth Muscle Contraction pathway. The abscissa represents the experimental samples; the ordinate represents differential genes. (F) Relative mRNA level of genes related to smooth muscle contraction. PVSMC were transfected with negative control (NC) or Kindlin-2 siRNA, Total RNA were isolated 72h after Kindlin-2siRNA interference. *p < 0.05**p < 0.01*** *p* <0.001. (G) Western blot analysis of PVSMC 72h after Kindlin-2siRNA interference. (H) Western blot analysis of related protein expression of colon in WT and iKO mice, 4 weeks after tamoxifen treatment. See Figure S6 for additional information.

It is known that Ca^2+^ influx regulates smooth muscle contraction, and the findings above identified a key role for Kindlin-2 in controlling smooth muscle contraction. We then asked whether links exist between Kindlin-2 and calcium-regulating proteins. To answer this question, we measured Ca^2+^ influx in SMCs, with or without Kindlin-2. Given that Angiotensin II serves an important role in raising blood pressure during hypotension by promoting vasoconstriction due to increased Ca^2+^ influx 47, 48, we examined the role of Kindlin-2 in Angiotensin II-induced Ca^2+^ influx, which was measured using Fluo-3AM. The results showed that Kindlin-2 depletion decreased the Ca^2+^ influx and that Kindlin-2 was required for Angiotensin II-induced Ca^2+^ influx in SMCs (Figure 7A-B).

To determine the molecular mechanism underlying Kindlin-2-dependent control of Ca^2+^ influx, we performed RNA sequencing (RNA-seq) analyses, with or without Kindlin-2, using mouse SMCs. RNA was extracted from sigmoid colonic SMCs obtained from iKO mice (n = 3) and WT mice (n = 3) at 8 weeks of age (following 2-week treatment with tamoxifen) and used for RNA-seq analysis. Kyoto Encyclopedia of Genes and Genomes (KEGG) pathway analysis of highly enriched pathways showed that cytokine-cytokine-receptor interactions, cell-adhesion molecules, extracellular matrix-receptor interactions, chemokine-signaling pathways, the PI3K-Akt-signaling pathway, apoptosis, and some inflammation-related pathways were upregulated in iKO smooth muscles (Figure 7C). In contrast, protein processing in the endoplasmic reticulum, cell cycle progression, and vascular smooth muscle contraction were downregulated (Figure 7D). Interestingly, these processes rely upon sufficient cytosolic Ca^2+^ concentrations. The heatmap summarizes the pathways highlighted in Figure 7D and shows that smooth muscle markers (such as contractile proteins and Ca^2+^-binding proteins) were downregulated (Figure 7E). Further, quantitative PCR (qPCR) and Western blot analysis confirmed that the Kindlin-2 knockdown in PVSMCs downregulated the SMC markers Acta2 and Myh11, the calcium channel protein Stim1, and S100A14 (Figure 7F-G). Similar results were obtained in iKO mice, in which SMC marker CNN was also drastically downregulated (Figure 7H). These findings suggested that Kindlin-2 controls Ca^2+^ influx by regulating the expression of calcium-regulatory proteins (including Stim1 and S100A14) in smooth muscles.

## Discussion

Kindlin-2 is extensively expressed in mesoderm-derived organs and tissues, including the heart, skeleton, kidneys, testes, and muscle tissues 33, and Kindlin-2 is highly expressed in smooth muscles. Differentiating myocytes lacking Kindlin-2 cannot elongate and fail to fuse into multinucleated myotubes 30. Kindlin-2 was previously shown to regulate the myogenic regulatory factor, myogenin via canonical Wnt/β-catenin signaling 24. Thus, we wondered what role Kindlin-2 serves in smooth muscle development. To investigate its role, we constructed two mouse models to specifically knock out Kindlin-2 in smooth muscles. One model enabled us to investigate the role of Kindlin-2 during embryonic mouse smooth muscle development, and the other model was an iKO mouse line that enabled us to pinpoint the role of Kindlin-2 in smooth muscles in adult mice. Thereby, we showed for the first time that Kindlin-2 depletion in embryonic smooth muscles causes embryonic death at E14.5 due to a failure in smooth muscle layer formation and apoptosis. The loss of Kindlin-2 in adult smooth muscles disrupted their normal structure and function and led to an impairment of smooth muscle contractility and intestinal obstruction that caused adult mice to die.

The depletion of the gene encoding murine Kindlin-2 resulted in embryonic lethality before cardiogenesis 26, 27. Kindlin-2 ablation in Prx1-expressing mesenchymal progenitor cells caused neonatal lethality, chondrodysplasia, and loss of the skull vault 49. We found that Kindlin-2 depletion in smooth muscles resulted in embryonic death at E14.5, demonstrating a new important role for Kindlin-2 in smooth muscle development. It was previously shown that vasculogenesis is critical for embryonic survival and subsequent organogenesis 35, 50. To determine whether Kindlin-2 loss in the endothelium or smooth muscles causes embryonic death, we also generated vascular endothelium-specific Kindlin-2 knockout mice using the Tie2-cre system, which targets endothelial cells. However, no Kindlin-2^fl/fl^;Tie2-cre+ embryos were found at E10.5, suggesting that mice with this genotype were all dead and absorbed. Considering previous data showing that whole-body Kindlin-2-knockout mice died at E7.5 27, we, therefore, postulated that the endothelial-specific Kindlin-2 knockout mice died at E7.5-9.5, earlier than the smooth muscle-specific Kindlin-2 knockout mice. Vascular SMCs originate from various mesodermal lineages, and mesenchymal stem cells (MSC) differentiate into mature SMCs at E12.5-13.5 1. These findings suggest that Kindlin-2 may be involved in regulating MSC differentiation into mature smooth muscle during embryonic development. Our RNA-seq analysis showed that the loss of Kindlin-2 downregulated the transcription factor Sox9 (Figure 7E), which can regulate α-SMA, SM22α, and Myh11 expression. This result helps explain why the loss of Kindlin-2 led to SMC marker downregulation (Figure 1D and Figure 7G-H).

The absence of Kindlin-2 in the smooth muscles of adult mice resulted in intestinal structural disorder and dysfunction, which leads to intestinal obstruction and the death of iKO mice. Vascular smooth muscle maintains vasoconstriction and decreased blood pressure (Figure 3D-E) in iKO mice reflects dysfunction of vascular smooth muscles. The decreased blood pressure may also result from late-stage gastrointestinal disease and reflect the individual's constitution. Therefore, we measured the tension of the vascular rings *ex vivo*, which provided more direct evidence that vasoconstriction in the Kindlin-2 iKO group decreased significantly. Our necropsy results (Figure 3G) combined with data showing that iKO mice displayed poor appetite and a longer whole-gut transit time showed that iKO mice died from severe intestinal obstruction, not vascular dysfunction.

Western blot analysis of colonic smooth muscle lysates showed that heterozygous depletion of Kindlin-2 led to downregulation of smooth muscle structural proteins (Figure 3I-J), which suggested that Kindlin-2 affected smooth muscle structural proteins in a dose-dependent manner, supporting a regulatory role of Kindlin-2 for smooth muscle structural proteins. In addition, heterozygous depletion of Kindlin-2 reduced the expression of these markers but did not significantly affect smooth muscle function *in vivo*, and only the iKO group, which caused a complete knockout of Kindlin-2, displayed a severe phenotype.

In the myograph experiments, we were unable to measure the diastolic function independently, and pre-contraction was determined as the baseline before measuring relaxation. In our study, smooth muscle contraction was seriously damaged in iKO mice (Figure 6A and C); thus, the relaxation measurements were not significant (Figure 6B and D). Loss of Kindlin-2 greatly accelerated smooth muscle dysfunction with concomitant loss of expression of the contractile-related proteins α-SMA, Myh11, CNN, and Stim1. Stim1 and Orai1 were previously identified as components of the store-operated Ca^2+^ channel 2. In this study, RNA-seq analysis showed that both Stim1 and Orai1 were downregulated in iKO mice (Figure 7B). These data indicate that Kindlin-2 is required for Stim1 expression and helps maintain Stim1-dependent store-operated Ca^2+^ influx, which further maintains SMC contractile function. Stim1 and Orai1-dependent store-operated Ca^2+^ influx are critical for VSMC proliferation and migration 51, which are the main characteristics of SMC phenotype switching. In addition, Angiotensin II can activate Ca^2+^ entry across the SMC plasma membrane through the Stim1-Orai1 pathway, and knockdown of Stim1 and Orai1 suppressed Ang II-mediated Ca^2+^ influx and cell proliferation in synthetic VSMCs 52. These findings explain why Kindlin-2 downregulation neither promoted SMC proliferation and migration (data not shown) nor changed the phenotype of SMCs.

**Figure 8:**
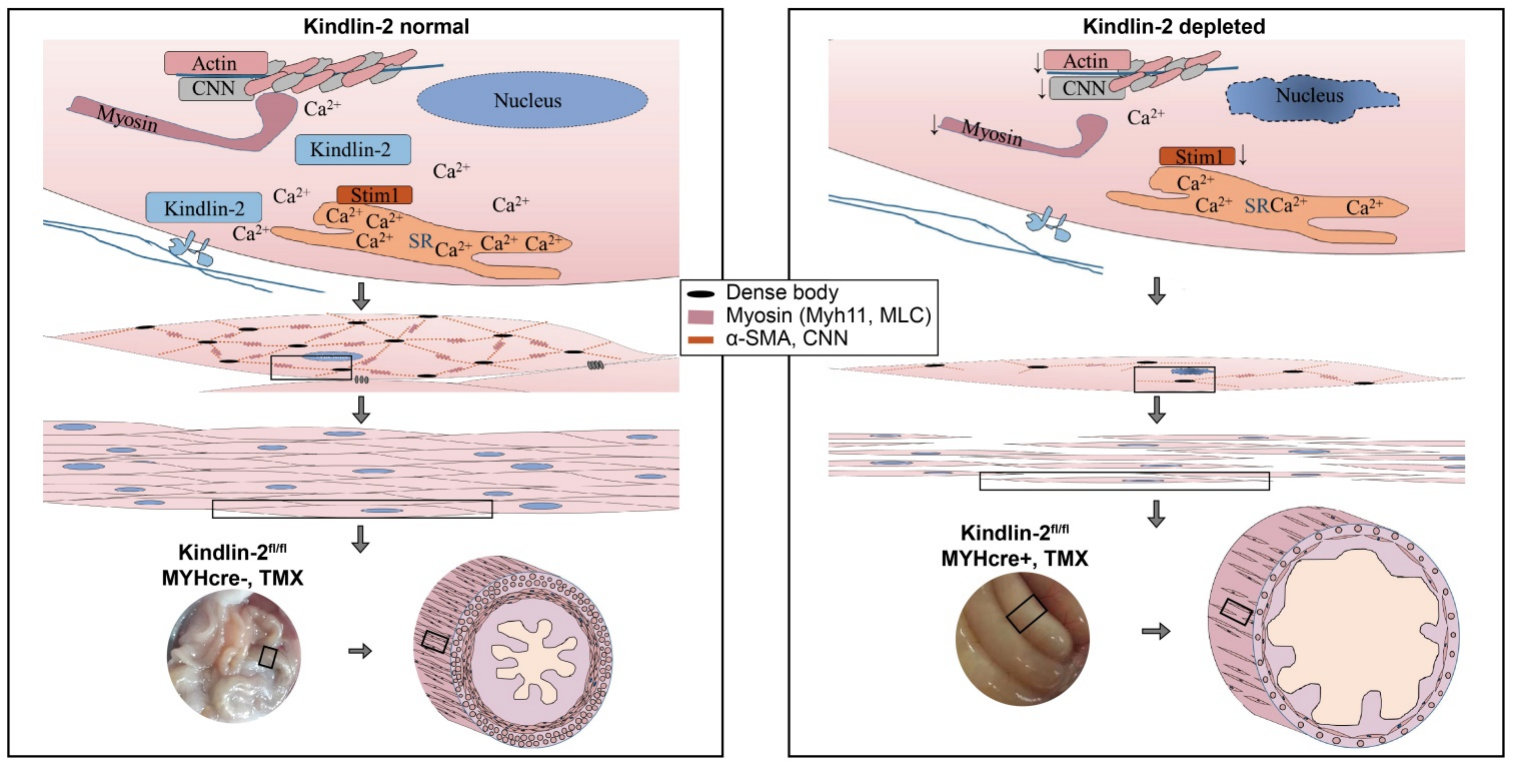
** A working model for Kindlin-2 depletion induces intestinal obstruction.** Loss of Kindlin-2 induces apoptosis of smooth muscles so that the number of smooth muscle cells decrease greatly. Kindlin-2 depletion induces loss of smooth muscle structural proteins and Ca^2+^ channel proteins, which destroying the existing smooth muscle cells' structure and contraction functions. As a result, the wall of gastrointestinal tract becomes thinner, the peristalsis ability decreases, the lumen expands and expands, which eventually leads to intestinal obstruction.

Based on the fact that Kindlin-2 is essential for smooth muscle structure and function, we established a working model that depicts the role of Kindlin-2 in smooth muscle (Figure 8). In this model, loss of Kindlin-2 induces apoptosis of smooth muscles so that the number of smooth muscle cells decreases greatly. Kindlin-2 depletion induces the downregulation of smooth muscle structural proteins and Ca^2+^ channel proteins, which destroy the structure of existing smooth muscle cells and contractile function. As a result, the wall of the gastrointestinal tract becomes thinner, the peristalsis ability decreases, the lumen expands drastically, which eventually leads to intestinal obstruction.

It is widely known that calcium overload can lead to increased mitochondrial membrane permeability, cytochrome calcium influx and oxidative stress, and also activate caspase 3-dependent apoptosis in SMC 53-55. Here we found that the decrease of calcium channel severely affects the calcium influx and the contraction of smooth muscle. However, it is rarely known that the decrease of calcium influx can cause apoptosis of SMCs, but we cannot rule out the possibility that Kindlin-2 might inhibit SMC apoptosis via stabilizing normal Ca^2+^ concentration, which is worth of further investigation. Given that Kindlin-2 is critical for integrin signaling through direct binding to the cytoplasmic tail of β integrins, and Kindlin-2 also functions as a coactivator for Talin-mediated integrin activation which promotes cell viability, adhesion and spreading 22. In addition, Kindlin-2 binds to components of the actin cytoskeleton 56, which is indispensable for SMC contractility. Together, depletion of Kindlin-2 results in inactivation of smooth muscle integrins, which further lead to SMC apoptosis. Further, in this study KEGG pathway analysis showed that the PI3K-Akt-signaling pathway were upregulated in iKO smooth muscles, and it was known that phosphorylation of Akt inhibits proliferation and promotes apoptosis in SMCs 57.

It should be noted that we found that the iKO phenotype was similar to that of patients with smooth muscle-motility disorders such as chronic intestinal pseudo-obstruction (CIPO) and megacystis microcolon intestinal-hypoperistalsis syndrome (MMIHS). CIPO may be congenital or acquired secondary to other disorders. One-third of congenital CIPO cases are due to smooth muscle myopathies, and proper diagnosis and treatment of CIPO are difficult because the underlying pathology is poorly understood 6, 7. MMIHS is a rare congenital disorder characterized by impaired smooth muscle contraction. Heterozygous mutations in MYH11 and ACTA2, and homozygous mutations in MYH11, MYLK, and LMOD1 are at the root of vascular smooth muscle dysfunction 12-15. Our findings also suggest that a defect in Kindlin-2 to MYH11 regulation may cause vascular smooth muscle dysfunction. Thus, it is worthy to identify potential Kindlin-2 mutations in patients that may link these diseases. Knockout of Kindlin-2 leading to apoptosis of smooth muscle cells in mice indirectly prove that Kindlin-2 contributes to SMC survival. Just as LncRNA GAS5 inhibiting apoptosis of SMC to become a strategy against abdominal aortic aneurysm (AAA) 57 and miR-27a targeting apoptosis of ECs and strongly diminishing occurrence of aortic dissection (AD) 58, Stabilizing Kindlin-2 might potentially serve as a novel strategy against CIPO. Therefore, Kindlin-2 iKO mice may serve as a suitable research model and contribute to testing therapeutics for these diseases.

## Supplementary Material

Supplementary figures and tables.

## Abbreviations

CIPO: chronic intestinal pseudo-obstruction
CNN: calponin
CTFM: cell traction force microscopy
ER: endoplasmic reticulum
FN: fibronectin
IP3: 1,4,5-trisphosphate
KEGG: Kyoto Encyclopedia of Genes and Genomes
KLF4: Kruppel like factor 4
L-NAME: NG-nitro-L-arginine
MLC20: 20-kDa regulatory myosin light chain
MLCK: myosin light chain kinase
MMIHS: megacystis microcolon intestinal-hypoperistalsis syndrome
MSC: mesenchymal stem cell
Myh11: myosin heavy chain 11
PCR: polymerase chain reaction
PDGF: Platelet-derived growth factor
PLCβ: phospholipase C β
PVSMC: primary vascular smooth muscle cell
SM22α: smooth muscle protein 22α
SMC: smooth muscle cell
SMMHC: smooth muscle myosin heavy chain
SOCC: store-operated Ca2+ channel
SOCE: store-operated calcium entry
SR: sarcoplasmic reticulum
α-SMA: smooth muscle α actin
