# Supplementary material for: Kindlin-2 deficiency induces fatal intestinal obstruction in mice
Source: Theranostics. 2020 May 15;10(14):6182–200. doi: 10.7150/thno.46553 (PMC7255029; doi:10.7150/thno.46553)
Supplement: Supplementary file 1 — Supplementary figures and tables. [file thnov10p6182s1.pdf]

Figure S1

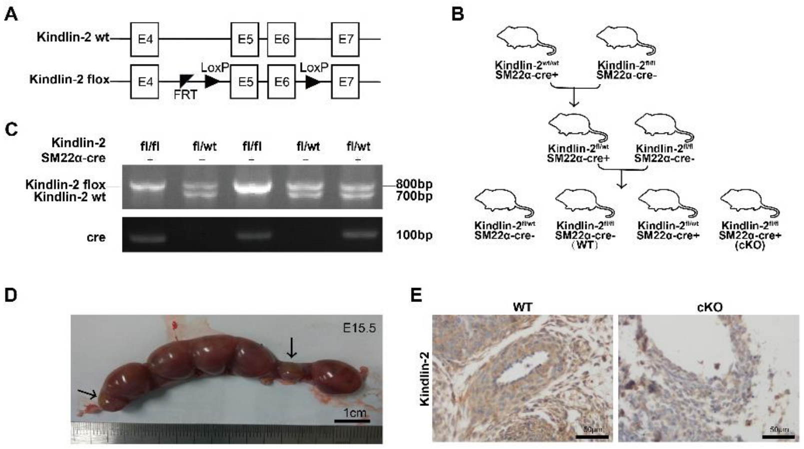

Figure S1. Generation and identification of smooth muscle specific Kindlin-2 depletion mice model

Related to Figure 1.

(A) A schematic of the relevant genomic region of Kindlin-2. The wild band (Kindlin-2 wt, up), and the franked locus (Kindlin-2 flox, below) is shown. Exons 5 and 6 of Kindlin-2 gene are flanked by loxP sites, a FRT site is remained as the floxed mice are derived from FRT knock-out-first mice; isosceles triangles indicate LoxP sites, and the rectangular triangle indicates FRT site. (B) Generation routine of smooth muscle (SM)-specific Kindlin-2 depletion mice. Kindlin-2 heterozygous depletion mice (Kindlin-2fl/wt, SM22α-cre+) were generated by cross-breeding Kindlin-2 floxed (Kindlin-2fl/fl) mice with SM22α-cre transgenic mice. Kindlin-2fl/wt, SM22α-cre+ mice backcrossed with Kindlin-2fl/fl mice to deliver SM-specific Kindlin-2 depletion mice. (C) Detection of mice genotype. DNA products were separated by size within a 2% agarose gel electrophoresis, The 800bp and 700bp bands represent Kindlin-2 floxed (Kindlin-2 flox) and wildtype (Kindlin-2 wt) alleles, respectively (up). Cre band is shown in the below panel. (D) Gross appearance of a uterus dissected from a 15.5d-pregnant female mouse, pregnancy was predicted using vagina plug. Arrows show arrested embryos. Scale bar, 1cm. (E) Immunohistochemistry of Kindlin-2 (brown), artery cross section at E12.5 were stained for testing Kindlin-2 protein level. Scale bar, 50μm.

Figure S2

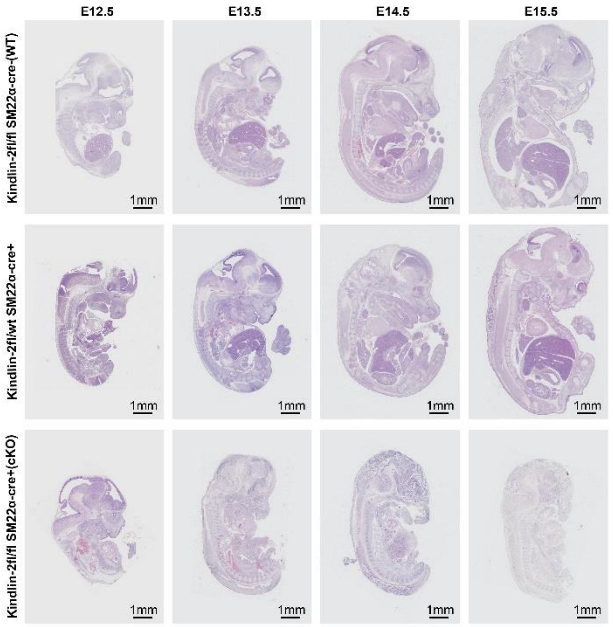

Figure S2. Heterozygous depletion of Kindlin-2 exhibits no significant phenotype change compared with wild type mice.

Related to Figure 1.

HE staining of the whole embryo sections at sagittal plane. The WT (upper), heterozygous littermates (Kindlin-2fl/wt, SM22α-cre+) and cKO mice (lower) were presented, time range from E12.5 to E14.5. Scale bar, 1mm

Figure S3

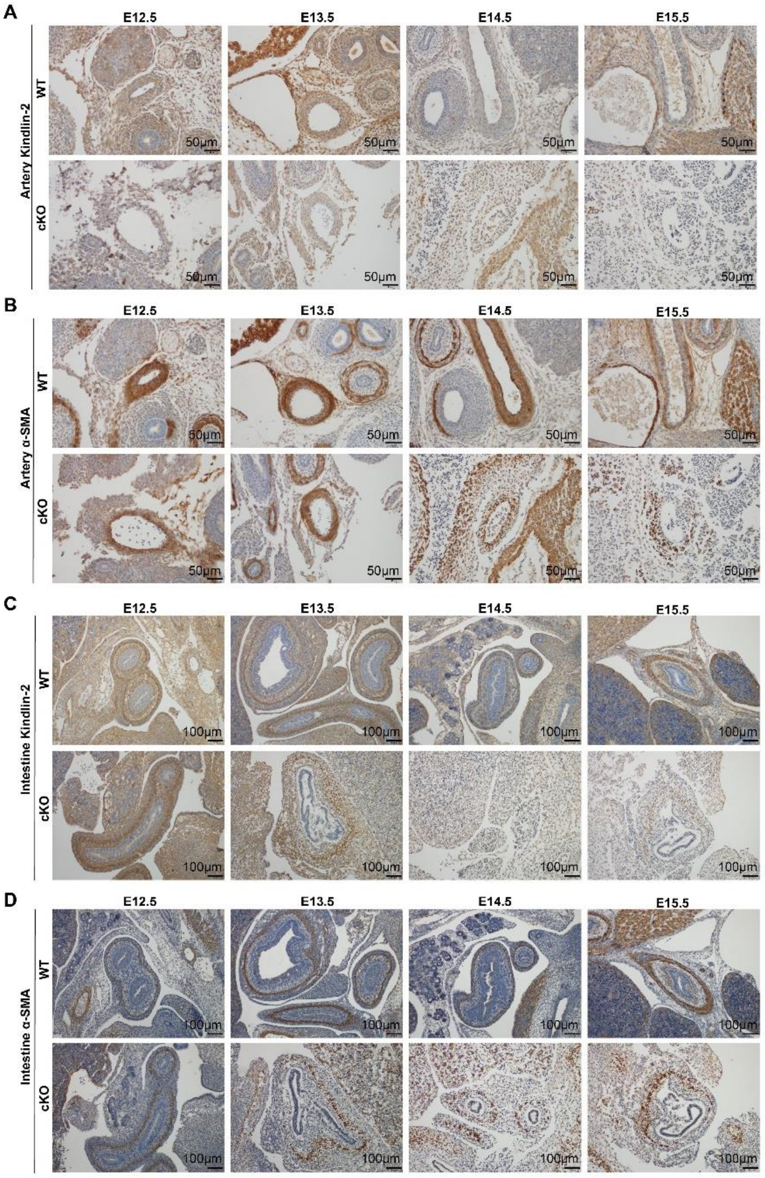

Fig S3. cKO embryos display a decreased trend of Kindlin-2 and α-SMA expression.

Related to Figure 2.

(A) Immunohistochemical staining of E12.5-15.5 WT and Kindlin-2 cKO embryonic sections for Kindlin-2 in arteries. Scale bar, 50μm. (B) Immunohistochemical staining of E12.5-15.5 WT and Kindlin-2 cKO embryonic sections for α-SMA in arteries. Scale bar, 50μm. (C) Immunohistochemical staining of E12.5-15.5 WT and Kindlin-2 cKO embryonic sections for Kindlin-2 in intestine. Scale bar, 100μm. (D) Immunohistochemical staining of E12.5-15.5 WT and Kindlin-2 cKO embryonic sections for α-SMA in intestine. Scale bar, 100μm.

Figure S4

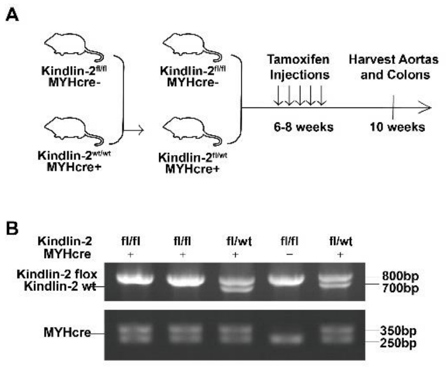

Figure S4. Generation and identification of conditionally specific knockout of Kindlin-2 in adult mice smooth muscle.

Related to Figure 3.

(A) Generation of Kindlin-2$^{fl/fl}$;MYHcre+ mice and procedure of Tamoxifen treatment. Kindlin-2 heterozygous (Kindlin-2$^{fl/wt}$;MYHcre+) mice were generated by cross-breeding Kindlin-2 floxed(Kindlin-2$^{fl/fl}$) mice with MYHcre transgenic mice, and backcrossed with Kindlin-2fl/fl mice to produce Kindlin-2$^{fl/fl}$;MYHcre+ mice. Mice were treated with tamoxifen via intraperitoneal injection starting at 6-8 weeks of age, following a 2-week rest period before other experiments. (B) Identification of mice genotype. DNA products were separated by size within a 2% agarose gel electrophoresis, The 800bp and 700bp bands represent Kindlin-2 floxed (Kindlin-2 flox) and wildtype (Kindlin-2 wt) alleles, respectively (up). In the below panel, two bands mean MYHcre positive; and a 250bp band means cre negative.

Figure S5

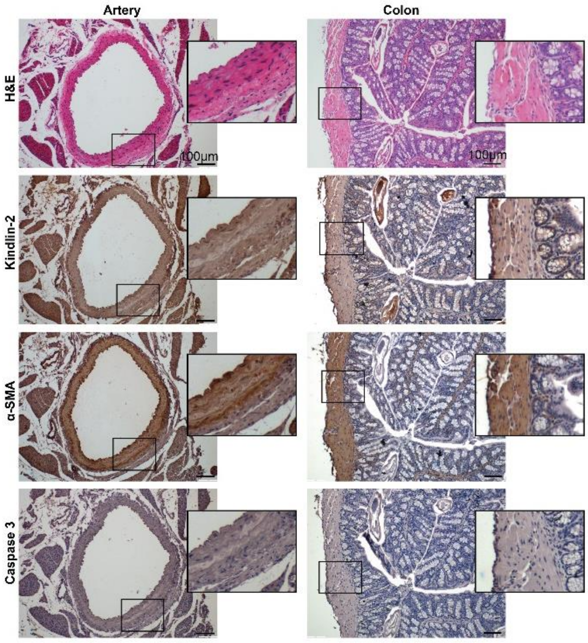

Figure S5. Heterozygous depletion of Kindlin-2 exhibits no significant changes of α-SMA and caspase3 level in smooth muscle tissues compared with wild type mice.

Related to Figure 4.

H&E and Kindlin-2, α-SMA and cleaved caspase 3 immunohistochemical staining of Kindlin-2fl/wt MYHcre+ aortas and colons. Mice were treated with tamoxifen injection at 8 weeks, and sigmoid colons were excised and fixed 14 days after injection. Scale bar, 100μm.

Table S1 Birth rate of Kindlin-2$^{fl/fl}$;SM22α-cre- × Kindlin-2$^{fl/wt}$;SM22α-cre+offsprings classified by genotype

Genotype	Gender	Number (rate)		Theoretical Mendelian ratio
Kindlin-2$^{fl/wt}$;SM22α-cre-	♂	26 (19.40%)	50 (37.31%)	25%
	♀	24 (17.91%)		
Kindlin-2$^{fl/fl}$;SM22α-cre-	♂	22 (16.42%)	43 (32.09%)	25%
	♀	21 (15.67%)		
Kindlin-2$^{fl/wt}$;SM22α-cre+	♂	20 (14.93%)	41 (30.60%)	25%
	♀	21 (15.67%)		
Kindlin-2$^{fl/fl}$;SM22α-cre+	♂	0 (0%)	0 (0%)	25%
	♀	0 (0%)		
Total	♂+♀	134 (100%)	134 (100%)	100%

Table S2

Statistics of embryonic development at indicated time point

day	E12.5		E13.5		E14.5		E15.5	
genotype	WT	cKO	WT	cKO	WT	cKO	WT	cKO
normal	19	5	21	2	31	0	32	0
abnormal	0	0	0	1	0	0	0	0
death	0	0	0	0	0	10	0	8

Table S3 Birth rate of Kindlin-2$^{fl/fl}$;Tie2-cre- × Kindlin-2$^{fl/wt}$;Tie2-cre+offsprings classified by genotype

Genotype	Gender	Number (rate)		Theoretical Mendelian ratio
Kindlin-2$^{fl/wt}$;Tie2-cre-	♂	16 (18.39%)	31 (35.63%)	25%
	♀	15 (17.24%)		
Kindlin-2$^{fl/fl}$;Tie2-cre-	♂	13 (14.94%)	29 (33.33%)	25%
	♀	16 (18.39%)		
Kindlin-2$^{fl/wt}$;Tie2-cre+	♂	12 (13.79%)	27 (31.03%)	25%
	♀	15 (17.24%)		
Kindlin-2$^{fl/fl}$;Tie2-cre+	♂	0 (0%)	0 (0%)	25%
	♀	0 (0%)		
Total	♂+♀	87(100%)	87(100%)	100%
